# 

**DOI:** 10.1192/bjb.2021.64

**Published:** 2022-08

**Authors:** Claire Hilton

**Affiliations:** Historian in Residence at the Royal College of Psychiatrists, London, UK. Email: historian@rcpsych.ac.uk

**Figure:**
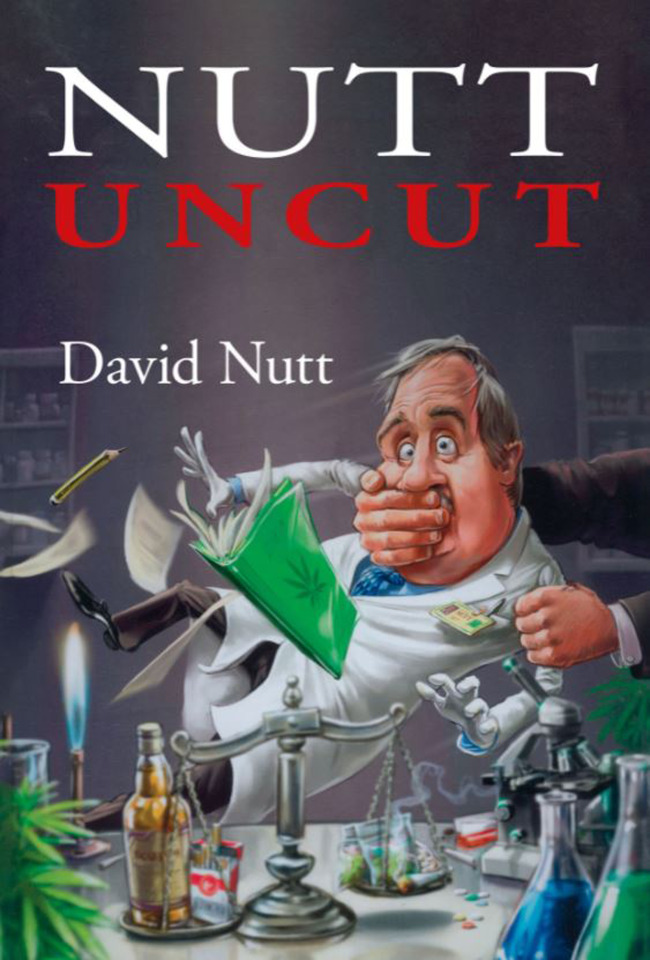

*Nutt Uncut* is a profile of psychopharmacology and healthcare policy development as much as an autobiography of Professor David Nutt. Regarding his desire to inform the public about the risks of psychoactive substances (legal and illegal), he has had the courage of his convictions to persevere with work in the field and to provide answers founded on research evidence.

Early in Nutt's career, anti-vivisection activists bombed his workplace and he was ‘put under the protection of the anti-terrorism police’. In 2009, he was sacked as chair of the UK's Advisory Council on the Misuse of Drugs, a statutory public body. The affair attracted widespread media attention. Nutt also described how the Cabinet Office deleted a chapter on the harm caused by alcohol from an independent report that it had commissioned and to which he had contributed. The Cabinet Office had taken advice from the drinks industry, a striking conflict of interest. Political, economic and vote-driven prejudices could supplant the best scientific evidence in policy-making. Nutt has encountered many other hurdles in his work. When planning to investigate the therapeutic effects of magic mushrooms (psilocybin) on depression, challenges included gaining research ethics committee approval, then obtaining the illegal drugs legally.

Politicians’ substance misuse mantra seems to be ‘once illegal, always illegal’. No drug has been removed from those listed as controlled substances in the Misuse of Drugs Act 1971, despite more recent research on relative harms. The Psychoactive Substances Act 2016, which Nutt designated ‘the most repressive piece of legislation in the UK for 400 years’, likewise marginalised risks. Misinformed international drug policy can also have untoward consequences: when the United Nations allowed police forces to seize the precursor of ecstasy, it resulted in black market trading of more hazardous alternatives.

Nutt's book achieves his goals, to ‘put into the public domain, in non-specialist terms, the truth about psychiatric disorders and their treatments’ and to counter ‘extreme and unfounded claims’ about drugs. It is also an absorbing read for clinicians who want to brush up on their psychopharmacology and to appreciate better the convoluted paths of government health policy decision-making.

